# Effects of Sequentially Removing Low-Abundance Taxon Records from Community Matrices on Aquatic Insect Community Evaluation in Mountain Streams

**DOI:** 10.3390/insects17101007

**Published:** 2026-09-30

**Authors:** Zhen Tian, Wei Zhao, Sicheng Ao, Xianfu Li, Qinghua Cai

**Affiliations:** 1 Institute of Hydrobiology, Chinese Academy of Sciences, Wuhan 430072, China; 2 College of Advanced Agricultural Sciences, University of Chinese Academy of Sciences, Beijing 101408, China; 3 State Key Laboratory of Regional and Urban Ecology, Zhejiang Key Laboratory of Pollution Control for Port-Petrochemical Industry, Ningbo Observation and Research Station, Institute of Urban Environment, Chinese Academy of Sciences, Xiamen 361021, China; 4 Institute of Eastern-Himalaya Biodiversity Research, Dali University, Dali 671003, China; 5 Center for Interdisciplinary Sciences, Dali University, Dali 671003, China

**Keywords:** mountain streams, aquatic insects, low-abundance taxa, community composition, community assembly

## Abstract

Low-abundance taxa are often filtered out of data sets before community analysis, which may remove information on both spatial community differences and associations between community composition and environmental gradients. We studied aquatic insect communities in mountain streams of the Hengduan Mountains, China, and sequentially removed low-abundance taxa from the lowest to the highest regional abundance. We compared community spatial heterogeneity, environmental and spatial associations, similarity between filtered and complete community matrices, and taxon-specific effects. Spatial turnover declined first, followed by abundance-based community dissimilarity. Associations of the community with environmental and spatial gradients were reorganized during removal, and compositional departure accelerated late in the sequence. The main effect dimension differed among taxa. Low abundance does not imply redundant ecological information: the uneven distribution of low-abundance taxon records contributes to estimated local distinctiveness and affects environmental and spatial associations as well as similarity to the complete community. Monitoring and community analyses in mountain streams should retain raw taxon records when possible, evaluate sensitivity to analytical filtering, and apply filtering rules suited to the study objective.

## Simple Summary

Low-abundance taxa are frequently overlooked in ecological data analyses because they are assumed to contribute little useful information. We tested this idea using aquatic insect data from streams in the Hengduan Mountains, China. We removed taxon records one by one from the observed community matrix, from the lowest to the highest regional abundance, and tracked how community metrics responded at each step. Taxon-composition differences among sites weakened first, while abundance-weighted differences declined only later; the associations between community composition and environmental and spatial gradients also shifted gradually. These results show that low-abundance taxon records contain information about local stream conditions and community composition that can be lost during analytical filtering. Low-abundance taxa should therefore not be removed without testing threshold sensitivity, and monitoring should retain raw taxon records when possible.

## 1. Introduction

Natural communities often have long-tailed abundance distributions: a few common taxa account for most individuals, whereas many others occur at low abundance [1,2,3]. Low abundance, however, does not necessarily indicate low ecological relevance. It may reflect a persistent local population, restricted habitat use, an occasional immigrant, or imperfect detection. Abundance- or occurrence-based filtering can reduce sparsity in community analyses, but a fixed threshold may remove spatially restricted taxa and affect estimates of richness, bioassessment, and ecological condition [4,5]. The key question is therefore not simply how many individuals a taxon contributes, but what information it carries for community comparisons and how that information changes when low-abundance taxon records are removed from the community matrix.

Aquatic insects provide a useful system for examining this issue. Their diverse life histories, feeding modes, respiratory traits, dispersal abilities, and habitat preferences link them to multiple aspects of stream conditions, including water temperature, current velocity, substrate, dissolved oxygen, and organic matter inputs [6,7]. Their relatively short generation times and established use in biomonitoring also make them informative indicators of stream ecological condition [8]. Some specialist taxa are confined to particular headwaters or microhabitats and can provide fine-scale habitat information even when they are locally or regionally uncommon [4,9]. Removing such taxa may therefore affect ecological interpretation beyond what their contribution to total abundance would suggest.

Mountain streams provide a clear spatial context for this question. Dendritic networks, elevational gradients, and longitudinal connectivity structure aquatic insect communities through interacting environmental and spatial processes [10,11]. Taxa with restricted distributions may be concentrated in headwaters, tributaries, or environmental transition zones. Their analytical removal could reduce differences among sites before it substantially changes abundance-weighted community structure. The consequences may also differ among components of beta diversity: turnover, nestedness, and abundance-based dissimilarity describe related but distinct aspects of community variation. Identifying when these components respond is important for interpreting the effects of low-abundance taxon removal.

Low-abundance taxon records may influence both the relationship between community composition and environmental or spatial gradients and the extent to which a filtered dataset represents the complete community. Community composition is shaped by environmental heterogeneity, spatial configuration, and their shared influence, while spatial structure may also capture unmeasured environmental variation [12,13,14,15]. The similarity of a filtered assemblage to the complete community indicates how much of the original composition is retained; we refer to this property as community compositional retention [16,17,18]. Existing studies often rely on a single filtering threshold or response metric and have focused largely on microbial communities [19,20,21]. It remains unclear how low-abundance taxon records shape different dimensions of community information in aquatic insect communities.

We therefore examined whether low-abundance aquatic insect taxa contribute to different dimensions of community information and whether their influence changes along a sequential-removal trajectory. Using communities from 54 mountain-stream sites in the Hengduan Mountains, we addressed four questions: (1) which components of spatial heterogeneity respond earliest; (2) whether environmental and spatial associations change along the sequential-removal trajectory; (3) whether compositional retention declines nonlinearly; and (4) whether taxa differ in effect strength and dominant effect dimension. We expected spatial turnover to respond before abundance-based dissimilarity and total Hellinger beta diversity, associations with environmental and spatial gradients to change along the sequential-removal trajectory, compositional retention to decline more rapidly later in the sequence, and taxon-specific effects to vary among taxa and between isolated and sequential removals.

## 2. Materials and Methods

### 2.1. Study Area and Sampling Sites

**Figure 1:**
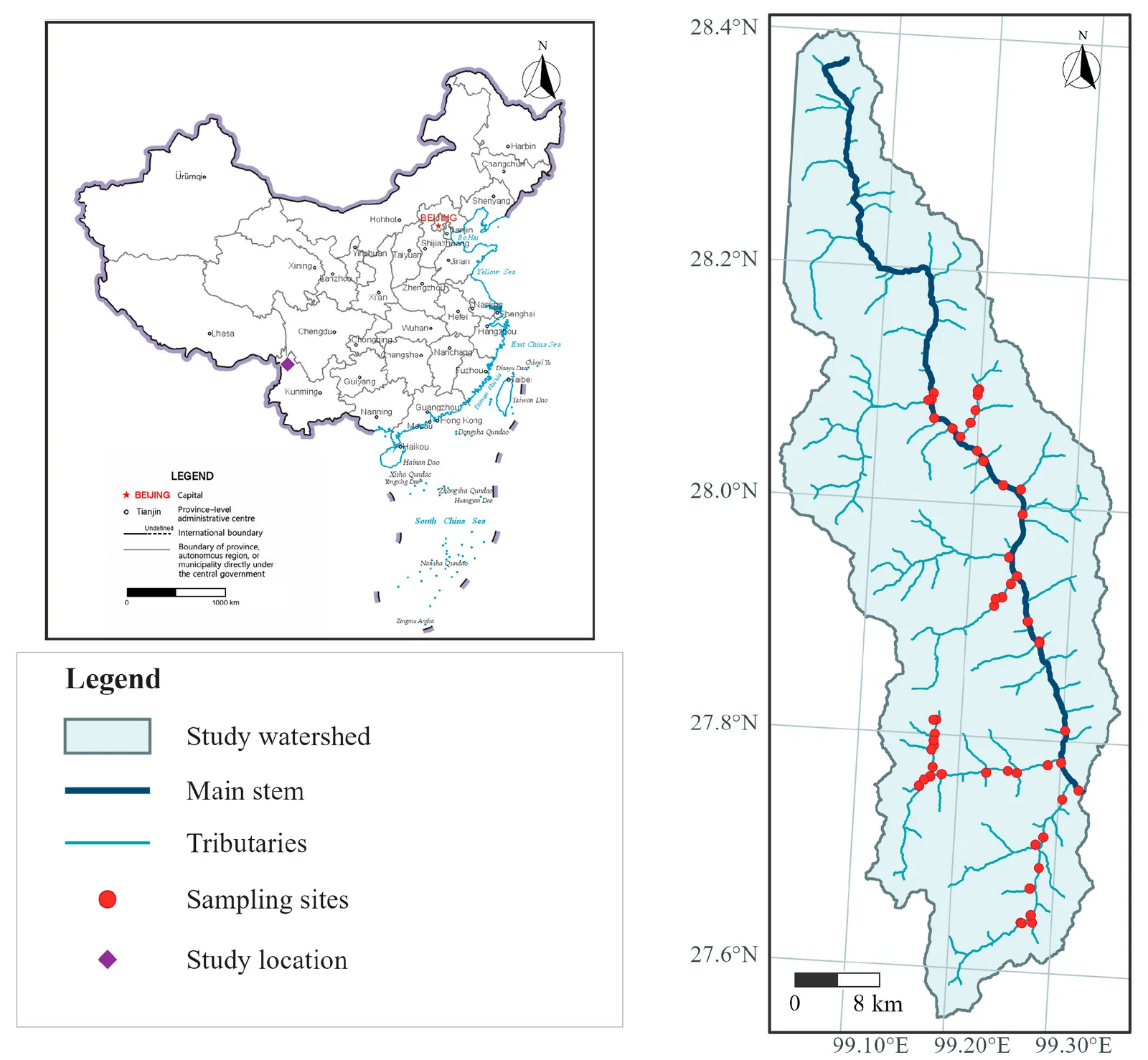
Location of the 54 mountain-stream sites in the study catchment on the western bank of the upper Jinsha River, central Hengduan Mountains, China. The dark channel is the DEM-derived main stem; lighter channels are tributaries. The inset shows the catchment location within China. Map approval number (Ministry of Natural Resources of China): GS(2019)1651.

The study area is a first-order tributary catchment on the western bank of the upper Jinsha River in the central Hengduan Mountains and is characterized by mountain-gorge streams. The regional climate is jointly influenced by the South Asian monsoon, the plateau monsoon, and topography. It has a cold-temperate montane monsoon climate with distinct wet and dry seasons: precipitation is concentrated in the warm season, winters and springs are dry, sunshine is strong, and the diurnal temperature range is large [22]. High relief and deep valley incision produce strong elevational differences in climate, vegetation, and hydrological processes: high-elevation reaches are cool and moist, whereas downstream valleys are warmer and drier. Steep slopes, dendritic networks, and coarse streambeds together form cold-water stream habitats and create strong local environmental heterogeneity among tributaries and between upstream and downstream reaches [23]. We established 54 sites along the main stem and tributaries between 27.642 and 28.099° N and between 99.150 and 99.319° E, at elevations of 2000–3130 m (Figure 1).

### 2.2. Community Sampling and Environmental Measurements

Field sampling was conducted in November 2017 during the regional dry season, and all 54 sites were sampled during the same survey campaign. At each site, five replicate Surber samples were distributed along a 100-m reach to represent the main habitat types present, including riffles and slower-flowing or pool habitats where available, as well as streambed substrates of different grain sizes. Benthic aquatic insects were collected with a 30 cm × 30 cm Surber sampler (sampling area, 0.09 m^2^; mesh size, 250 μm), which was held firmly against the streambed with its opening facing upstream. Substrates within the frame, including stones, gravel, cobbles, and finer particles, were disturbed, and stones were brushed so that dislodged organisms entered the net. The five replicate samples from each site were combined into a single composite sample before sorting and identification, giving a total sampled area of 0.45 m^2^ per site. Samples were rinsed through a sieve and transferred to labelled bottles containing 95% ethanol. In the laboratory, organisms were sorted and identified to the lowest reliable taxonomic level using larval morphology and standard identification keys [6,7]. Taxonomic assignments were standardized across sites by applying the same identification criteria; when species-level identification was not reliable, specimens were retained at the lowest confident level, including genus- or higher-level assignments. The resulting matrix therefore combines taxa at different taxonomic resolutions, which may influence abundance rankings and beta-diversity estimates; all analyses are interpreted as taxon-level rather than species-level patterns. Individuals were counted by site to produce an abundance matrix of 83 taxa (Table S1). Because low recorded abundance may reflect imperfect detection and seasonal phenology, the sequential-removal results are interpreted as taxon-level patterns under the observed sampling design rather than direct evidence of absence or temporal population change.

Coordinates and elevation were recorded at each site. Stream width was measured with a measuring tape or rangefinder. Water depth and current velocity were measured with a flow probe (FP101, Global Water Instrumentation, Inc., Gold River, CA, USA) at three cross-sections within the sampled reach. Several measurement positions were established at each cross-section, and current velocity was measured at 0.6 times the local water depth. Site-level current velocity was calculated as the arithmetic mean of all measured velocities. Air temperature was recorded at each site. Water temperature, pH, dissolved oxygen, salinity, electrical conductivity, oxidation–reduction potential, turbidity, and total dissolved solids were measured in situ with a handheld multiparameter water-quality meter (YSI 6600, YSI Incorporated, Yellow Springs, OH, USA). For laboratory water-chemistry analyses, approximately 100 mL of water was collected at each site in polyethylene bottles that had been rinsed with the sample water. Samples were acidified in the field with concentrated sulfuric acid to pH < 2 and transported to the laboratory under cooled, light-protected conditions. Dissolved organic carbon, total nitrogen, total phosphorus, ammonium nitrogen, nitrate nitrogen, and phosphate were measured with a continuous-flow analyzer (SAN++, Skalar Analytical B.V., Breda, The Netherlands). Periphyton chlorophyll a was measured from biofilms attached to streambed stones. Five stones were randomly selected within the sampled reach at each site. A circular cover with a radius of 27 mm was placed on the upper surface of each stone. The biofilm around the cover was removed with a nylon brush and rinsed away with stream water, whereas the area delimited by the cover was brushed with a soft brush and rinsed with distilled water into a 355-mL polyethylene bottle. A 100-mL aliquot was filtered through a glass-fiber filter (Whatman GF/C) and stored at −20 °C in the dark until analysis. Chlorophyll a was extracted with 90% acetone for 24 h and quantified using a spectrophotometer (UV-1601, Shimadzu Corporation, Kyoto, Japan). The verified accuracy specifications for the YSI 6600 were ±0.15 °C for water temperature, ±0.2 pH unit for pH, ±20 mV for oxidation–reduction potential, ±(0.5% of reading + 1 μS/cm) for electrical conductivity, ±max(1% of reading, 0.1 ppt) for salinity, ±max(2% of reading, 0.3 NTU) for turbidity, and ±max(1% of reading, 0.01 mg/L) for dissolved oxygen. Indicative detection limits for continuous-flow nutrient analyses were 0.01 mg N/L for total nitrogen, 0.005 mg P/L for total phosphorus, 0.0001–0.002 mg N/L for nitrate nitrogen, 0.0001–0.005 mg N/L for ammonium nitrogen, and 0.0001–0.002 mg P/L for phosphate. The indicative detection limit for dissolved organic carbon was approximately 0.1 mg C/L.

### 2.3. Sequential Removal Simulation

The complete community was represented by the abundance matrix of 54 sites × 83 taxa. Regional relative abundance was defined as the total number of individuals of a taxon across all sites divided by the total number of individuals in the community. Occurrence frequency was the number of sites at which a taxon was detected. Taxa with regional relative abundance below 2% were ordered from the lowest to the highest total abundance. Priority was therefore determined by increasing regional total abundance, representing the common analytical practice of filtering the least-abundant taxon records first. Ties were resolved first by occurrence frequency and then by taxon name. The 2% threshold defined the candidate taxa for sequential removal. At each step, the abundance of one taxon’s record was set to zero at all sites and all metrics were recalculated. This deterministic procedure evaluates the consequences of analytical filtering of the observed community matrix; it does not represent observed extinction, temporal community change, or response to a specific disturbance.

### 2.4. Spatial Heterogeneity Metrics

At each removal step, mean Bray–Curtis dissimilarity among the 54 sites was calculated with the vegdist function in vegan 2.6–10 [24,25]. After the community matrix was converted to presence-absence data, Sørensen dissimilarity was partitioned into the spatial turnover component *β*_sim_ and the nestedness component *β*_sne_ using the beta.pair function in betapart 1.6 [26,27]. The community matrix was Hellinger-transformed with the decostand function in vegan, and the sum of variances across taxa was used as total community beta diversity [28,29].

### 2.5. Analysis of Environmental and Spatial Associations

The Hellinger-transformed community matrix was used as the response matrix. The environmental model included elevation, dissolved oxygen, stream width, redox potential, and electrical conductivity. Environmental variables with non-negative values and sample skewness > 1 were log(1 + *x*)-transformed and then standardized. These five environmental variables were held constant across all removal steps. Coordinates were converted to a local planar system, and distance-based Moran eigenvector map (dbMEM) variables were generated with the dbmem function in adespatial 0.3–28 [30]. The six dbMEM variables denoted MEM1–MEM6 were held fixed across all removal steps; spatial predictors were not reselected after each removal. The truncation distance was the minimum distance required to connect all sites. Environmental, spatial, and combined models were fitted with the rda and RsquareAdj functions in vegan. Adjusted *R*^2^ was decomposed into pure environmental, pure spatial, shared, and unexplained fractions [12,15,25]. The pure spatial fraction represents community association with spatial structure not shared with the environmental variables.

### 2.6. Analysis of Community Compositional Retention

We defined a study-specific compositional similarity index (CSI) as one minus the Bray–Curtis dissimilarity [24] between each site’s complete taxon-abundance vector and its vector after sequential removal. Cumulative CSI for site *i* after removal step *k* was defined as follows:

$${\mathrm{CSI}}_{i,k}=1-\frac{\sum_{j=1}^{S}\left|x_{\mathrm{ij}}^{\left(0\right)}-x_{\mathrm{ij}}^{\left(k\right)}\right|}{\sum_{j=1}^{S}\left(x_{\mathrm{ij}}^{\left(0\right)}+x_{\mathrm{ij}}^{\left(k\right)}\right)}.$$

where S is the number of taxa in the complete community, and the abundance terms denote the observed counts of each taxon at site i in the complete matrix and after removal step k, respectively. Counts were not standardized; removal set the selected taxon counts to zero. CSI ranges from 0 (no abundance retained) to 1 (identical abundance vectors). A higher CSI indicates that the filtered assemblage is more similar to the complete assemblage. CSI was calculated separately for each site and removal step and summarized by its mean and the 10th, 25th, 50th, 75th, and 90th percentiles. It measures compositional retention under a deterministic filtering scenario, not temporal stability, resistance, or resilience.

### 2.7. Response Thresholds and Taxon-Specific Effects

Linear models and continuous single-breakpoint segmented linear models were fitted separately to the trajectories of community spatial heterogeneity, model-adjusted *R*^2^ values, and cumulative CSI. Candidate breakpoints were restricted so that at least 10 removal positions remained on each side. A breakpoint was considered supported when ΔBIC = BIC(linear) − BIC(segmented) was at least 6 [31]. For supported breakpoints with stable bootstrap distributions, conditional percentile 95% intervals were estimated from a cyclic moving-block residual bootstrap with a block length of 5 and 999 replicates. These intervals describe uncertainty in the fitted trajectory conditional on the observed data and fixed removal order, not field-sampling uncertainty. The isolated effect of a taxon was the change in a metric after that taxon alone was removed from the complete community; its sequential marginal effect was the change before and after it was removed within the fixed sequence. The compositional effect was the sum of absolute changes in mean Bray–Curtis dissimilarity, total Hellinger beta diversity, *β*_sim_, and *β*_sne_. The environmental-spatial effect was the sum of absolute changes in the pure environmental, pure spatial, shared, and unexplained fractions. These effects were converted to within-scenario percentiles. The combined taxon-specific sensitivity score was the arithmetic mean of the CSI, composition, and environmental/spatial percentiles; the eight highest-scoring taxa are displayed in the eight highest-scoring taxa were retained for visualization. The association between taxon occurrence percentage and isolated CSI loss was tested with a two-sided Spearman rank correlation; the association was specified as a single test, so no multiple-testing adjustment was applied. All analyses were implemented in R using base R, vegan, betapart, adespatial, and segmented.

### 2.8. Software and Visualization

All analyses were conducted in R 4.5.1 [32]. Data were organized with dplyr 1.1.4 and tidyr 1.3.1 [33,34]. Figures were produced with ggplot2 3.5.2, ggrepel 0.9.6, patchwork 1.3.0, and scales 1.4.0 [35,36,37,38].

## 3. Results

### 3.1. Overview of the Aquatic Insect Community

Across the 54 sites, we recorded 32,900 aquatic insect individuals belonging to 83 taxa. Site abundance, defined as the total number of individuals counted per sampled site, ranged from 155 to 1558 individuals (609.3 ± 314.5, mean ± SD), and taxon richness ranged from 16 to 50 (36.2 ± 7.2). Site abundance was positively correlated with taxon richness (Spearman *ρ* = 0.632, *n* = 54, *p* < 0.001).

**Figure 2:**
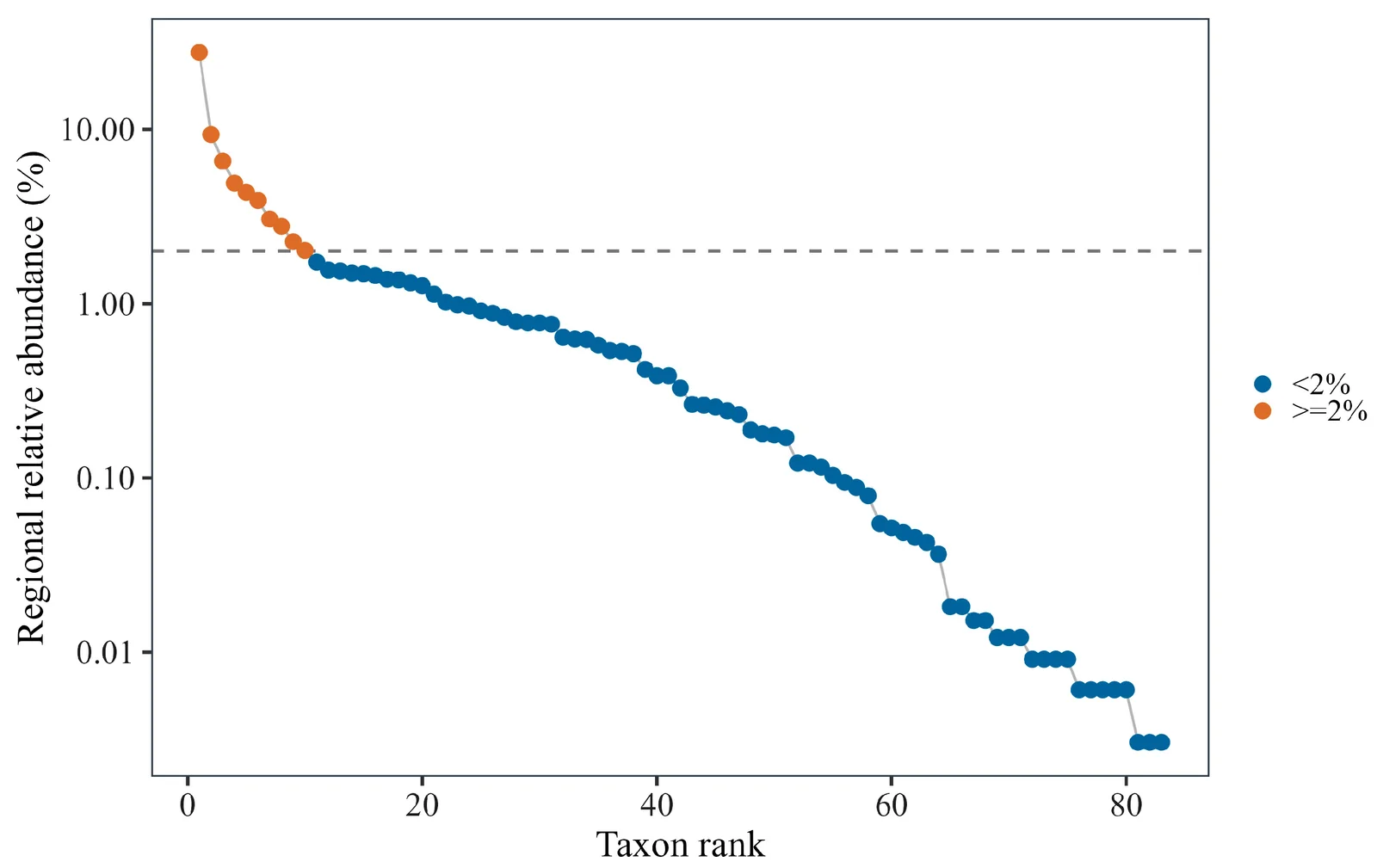
Regional rank-abundance distribution of the aquatic insect community. The regional community contained 83 taxa, of which 73 had regional relative abundance below 2%. The dashed line marks the 2% regional relative-abundance threshold; point colors distinguish candidate low-abundance taxa (<2%) from taxa at or above the threshold (≥2%). Taxon labels are omitted from the plot; standardized taxon names and ranks are listed in the taxonomy worksheet of Table S1.

The community showed a clear long-tailed distribution (Figure 2). The 73 taxa with regional relative abundance below 2% represented 88.0% of all taxa and 33.16% of all individuals; 21 of these taxa occurred at no more than five sites. Diptera and Ephemeroptera accounted for 39.7% and 29.8% of all individuals, respectively. Trichoptera accounted for 9.9% of individuals and included 26.5% of all taxa.

### 3.2. Sequential Removal and Spatial Heterogeneity

**Figure 3:**
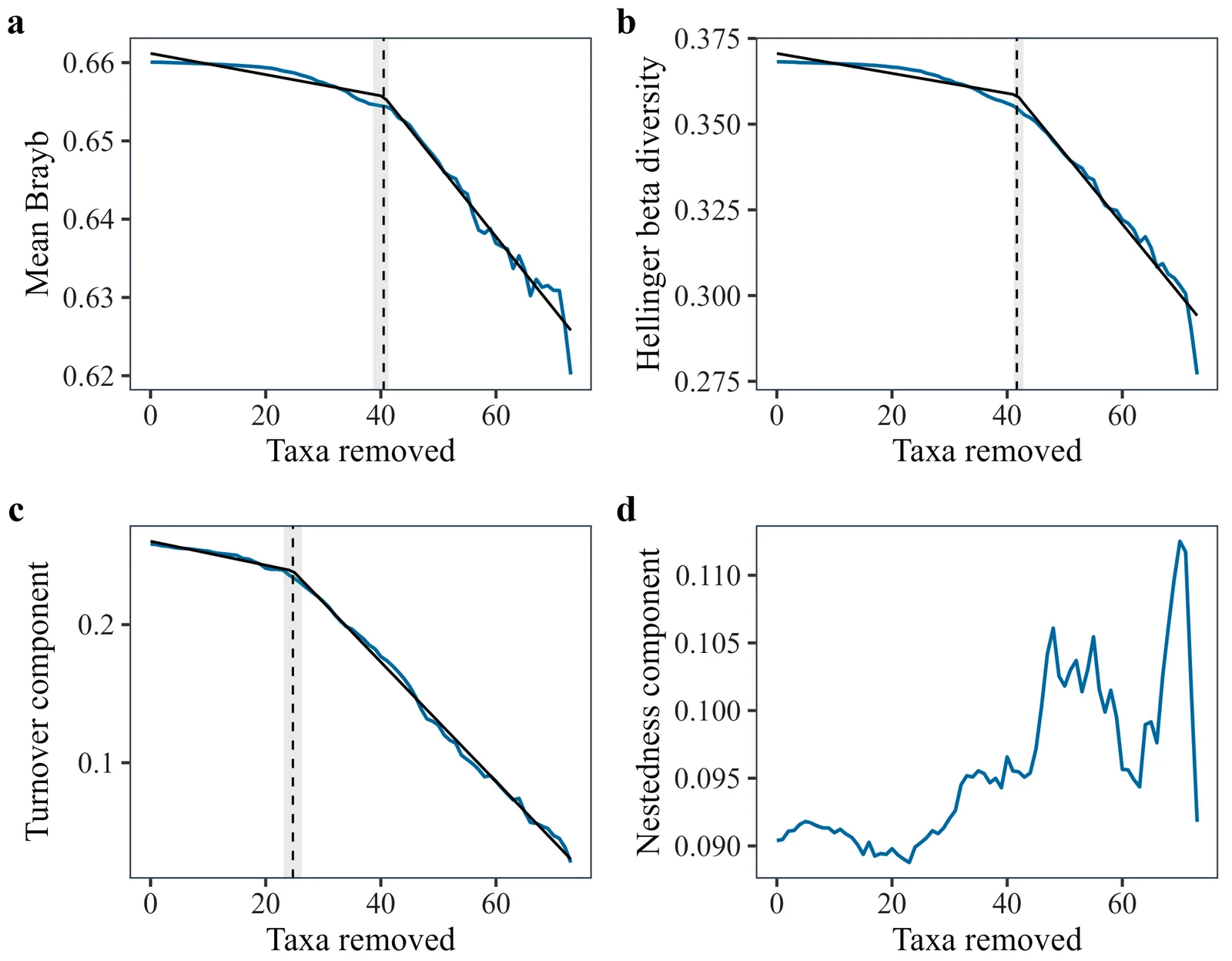
Community spatial heterogeneity during sequential removal. (**a**) Mean Bray–Curtis dissimilarity; (**b**) total Hellinger beta diversity; (**c**) spatial turnover component *β*_sim_; and (**d**) nestedness component *β*_sne_. Observed trajectories are shown as blue lines. Black lines are fitted segmented models for trajectories with ΔBIC ≥ 6; dashed vertical lines and light bands show supported breakpoint estimates and 95% confidence intervals. No breakpoint was supported for βsne because its segmented model did not improve sufficiently over the linear model.

After all 73 candidate taxa were sequentially removed, their cumulative abundance represented 33.16% of total abundance. Mean Bray–Curtis dissimilarity and total Hellinger beta diversity both declined during removal, with supported breakpoints at removal positions 40 (estimate 40.49, 95% CI 38.63–41.32) and 42 (estimate 41.68, 95% CI 41.17–42.79), respectively (Figure 3a,b; Table S2a). At these positions, the cumulative removals comprised 40 and 42 taxa (48.2% and 50.6% of all taxa), 950 and 1145 individuals (2.89% and 3.48% of all individuals), and 337 and 397 taxon-by-site occurrences (17.3% and 20.3% of all occurrences), respectively.

The spatial turnover component *β*_sim_ decreased from 0.258 to 0.028 and showed the earliest supported breakpoint at removal position 25 (estimate 24.72, 95% CI 23.09–26.25), when cumulative removal comprised 25 taxa (30.1% of all taxa), 151 individuals (0.46% of all individuals), and 84 taxon-by-site occurrences (4.3% of all occurrences). No supported breakpoint was detected for the nestedness component *β*_sne_ (ΔBIC = 1.42; Figure 3c,d).

### 3.3. Environmental and Spatial Associations

**Figure 4:**
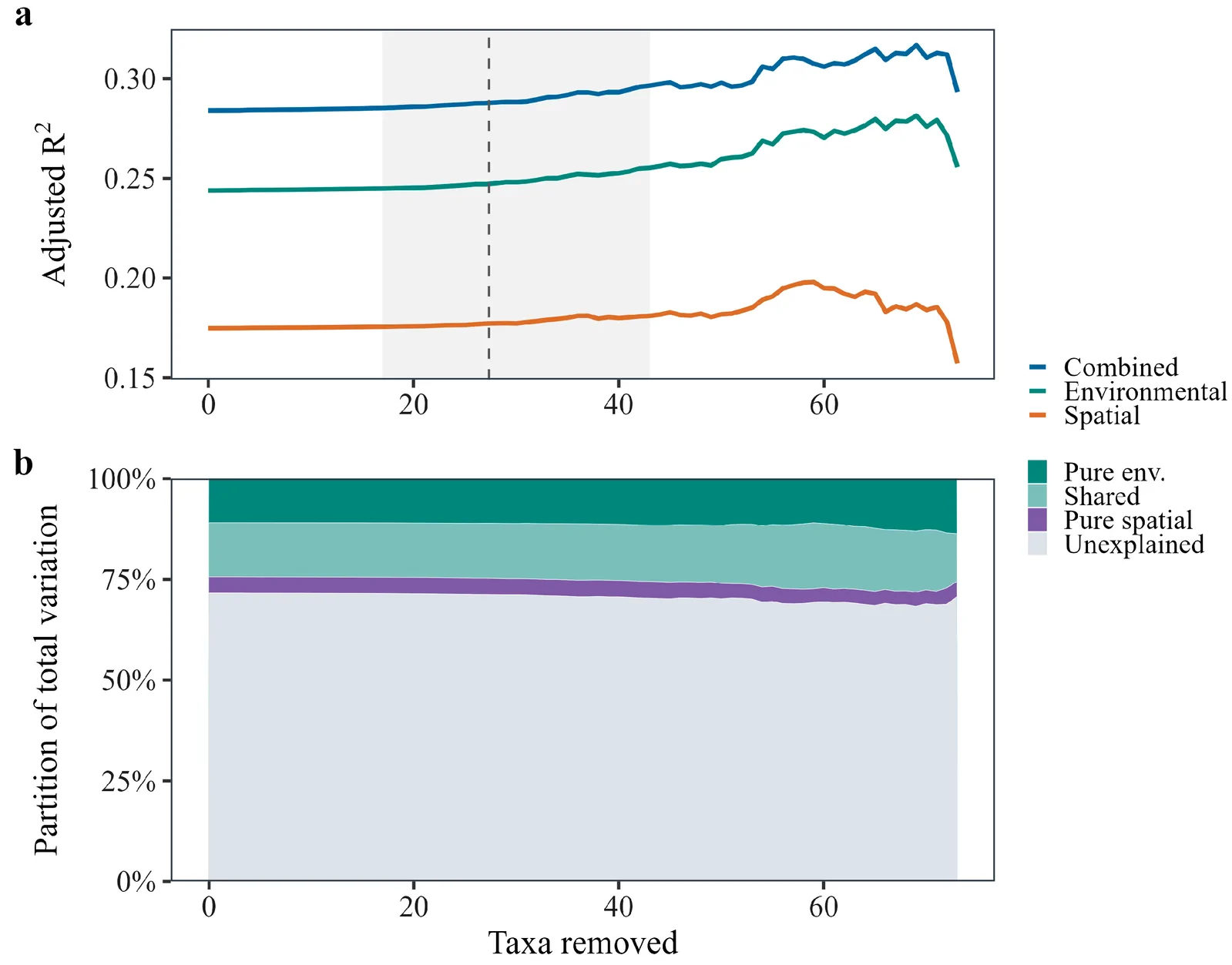
Environmental and spatial associations during sequential removal. (**a**) Adjusted *R*^2^ for the environmental, spatial, and combined models. (**b**) Variation-partitioning fractions for pure environmental, shared, pure spatial, and unexplained components. Lines show model estimates at each removal step; the dashed line and shaded band mark the combined-model breakpoint estimate and its 95% bootstrap interval.

The environmental model used five fixed variables (elevation, dissolved oxygen, stream width, redox potential, and electrical conductivity) and used six distance-based Moran eigenvector map (dbMEM) variables (MEM1–MEM6) as a fixed spatial-variable set. Throughout sequential removal, adjusted *R*^2^ was consistently higher for the combined model than for either the environmental or spatial model alone, and was generally higher for the environmental model than for the spatial model (Figure 4).

Adjusted R^2^ increased from 0.2841 to 0.2934 for the combined model and from 0.2439 to 0.2557 for the environmental model, while decreasing from 0.1748 to 0.1571 for the spatial model. The combined model showed a supported breakpoint at removal position 27 (estimated breakpoint 27.35, 95% bootstrap CI 17.00–43.01, ΔBIC = 25.28; Table S2b). At removal position 27, the cumulative removals comprised 27 taxa (32.5% of all taxa), 206 individuals (0.63% of all individuals), and 105 taxon-by-site occurrences (5.4% of all occurrences). Variation partitioning also changed: the pure environmental fraction increased from 0.1093 to 0.1363, the shared fraction decreased from 0.1346 to 0.1194, the pure spatial fraction decreased from 0.0402 to 0.0377, and the unexplained fraction decreased from 0.7159 to 0.7066 (Figure 4; Table S2b).

### 3.4. Community Compositional Retention

**Figure 5:**
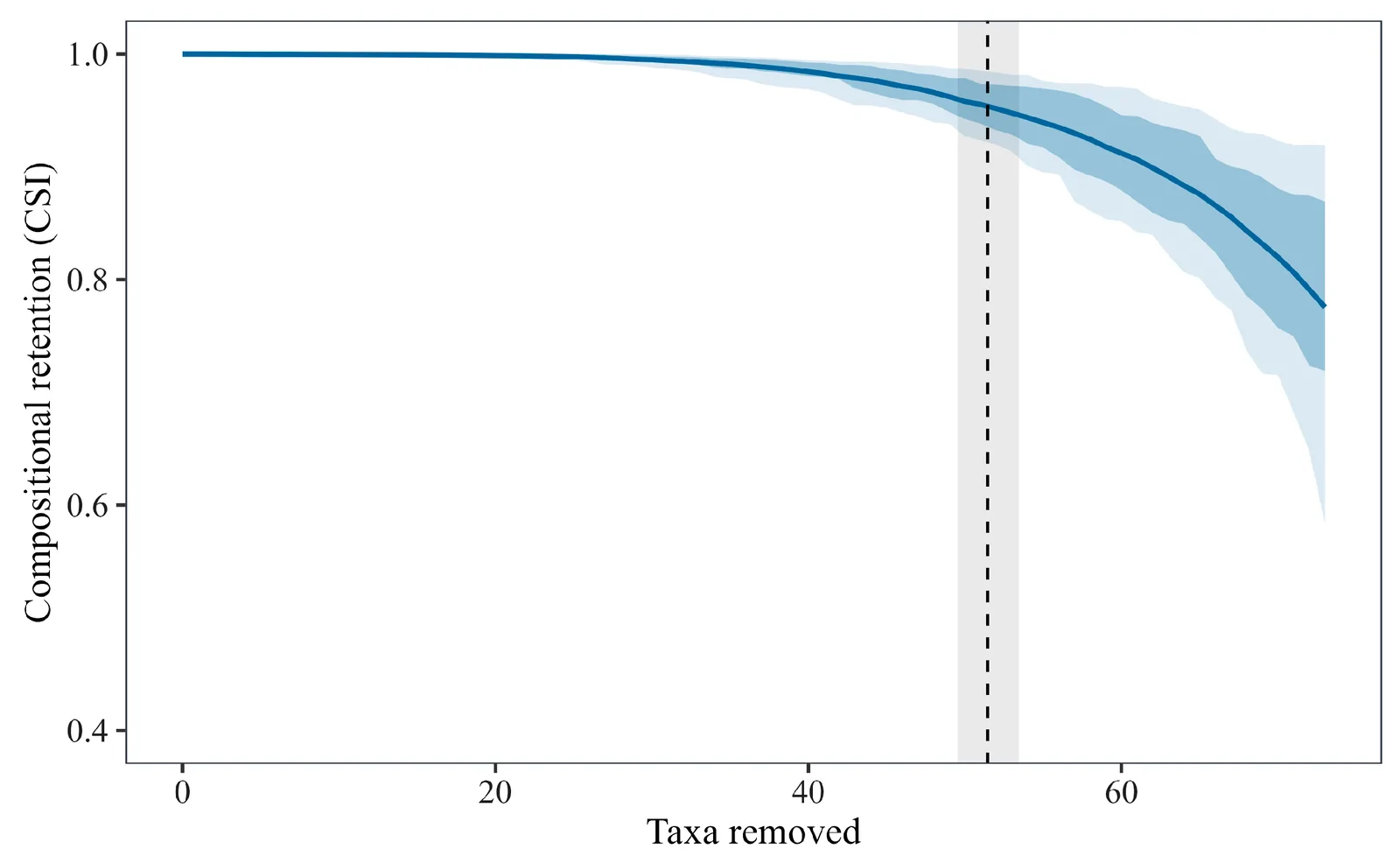
Community compositional retention during sequential removal. The curve shows mean cumulative CSI; dark and light ribbons show the 25–75th and 10–90th percentile ranges among sites, respectively. The dashed line and light vertical band show the supported CSI breakpoint and its 95% confidence interval.

Cumulative CSI declined more rapidly after the supported breakpoint at removal position 51 (estimated breakpoint 51.44, 95% CI 49.56–53.45, ΔBIC = 197.31; Figure 5). At removal position 51, the cumulative removals comprised 51 taxa (61.4% of all taxa), 2660 individuals (8.09% of all individuals), and 639 taxon-by-site occurrences (32.7% of all occurrences). Before this point, community composition remained very similar to the complete community; continued removal of taxa with higher abundance then produced substantially greater cumulative departure from the complete community. Mean CSI decreased from 1.000 at the complete community to 0.776 after all candidate taxa had been removed (Table S2c).

Differences among sites increased late in the removal sequence. The among-site standard deviation of cumulative CSI increased from approximately 0.024 at removal position 51 to 0.127 at the final step, where values ranged from approximately 0.450 to 0.967.

### 3.5. Taxon-Specific Effects

**Figure 6:**
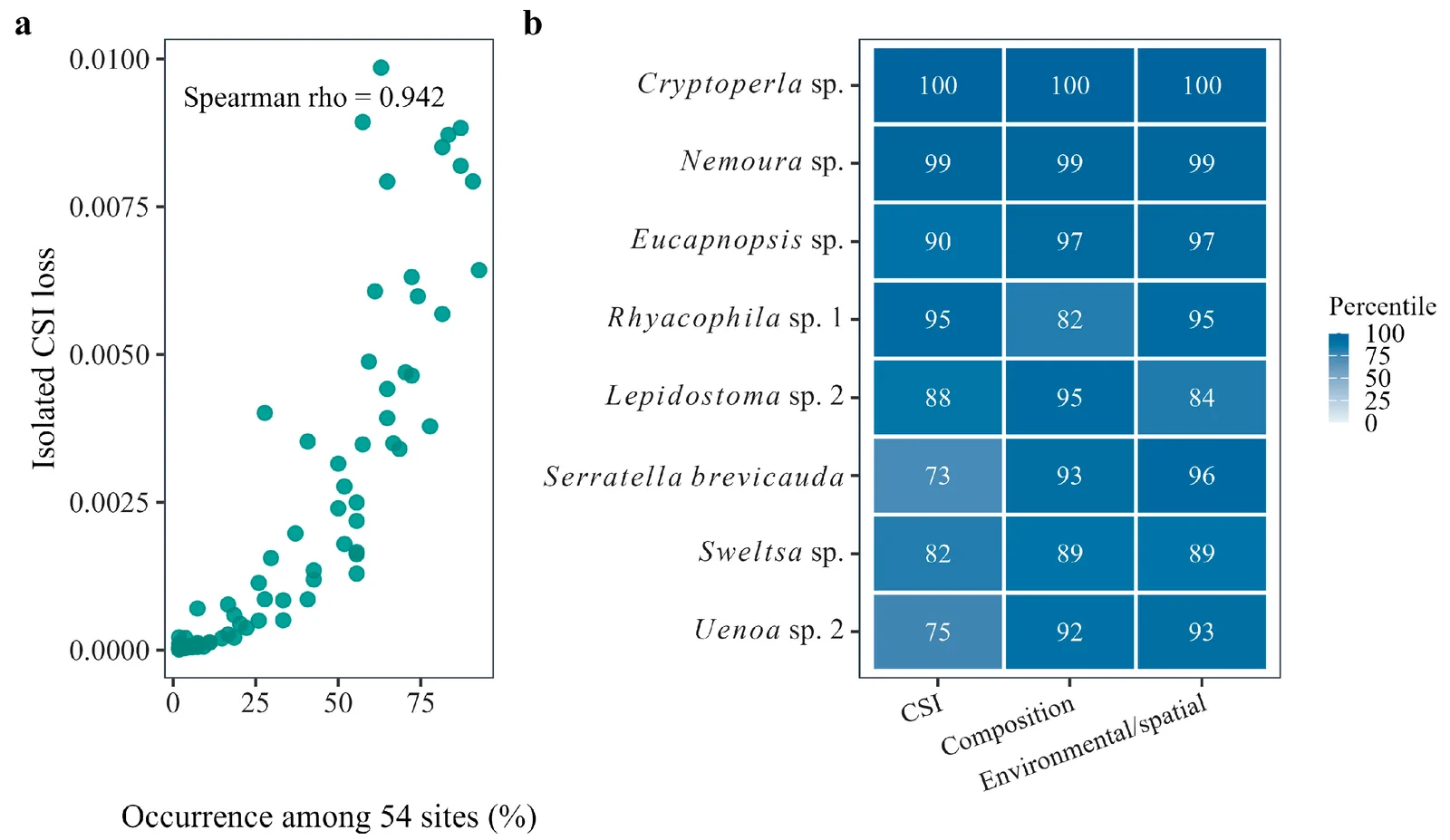
Taxon-specific effects under sequential removal. (**a**) Isolated CSI loss versus taxon occurrence percentage among the 54 sites. (**b**) Heatmap of the within-scenario effect percentiles for the eight taxa with the highest combined taxon-specific sensitivity scores across CSI, composition, and environmental/spatial effects. Panel (**b**) displays the standardized names directly; genus names and species epithets are italicized, whereas “sp.” and its numerical qualifiers are upright. The same eight taxa and their ranking are listed in Table S2d.

Taxon occurrence percentage was strongly correlated with isolated CSI loss (Spearman *ρ* = 0.942, *n* = 73, *p* < 0.001; Figure 6a; Table S2d). *Cryptoperla* sp. had the highest combined taxon-specific sensitivity score, followed by *Nemoura* sp. and *Eucapnopsis* sp. The eight taxa with the highest combined scores are shown by their standardized taxon names in Figure 6b and listed in Table S2d.

The top-ranked taxa showed different profiles across the three effect dimensions. *Cryptoperla* sp. and *Nemoura* sp. ranked highly across CSI, composition, and environmental/spatial effects, whereas other taxa reached their highest percentile in only one or two dimensions (Figure 6b; Table S2d).

## 4. Discussion

### 4.1. Effects of Low-Abundance Records on Estimated Spatial Turnover

Spatial turnover *β*_sim_ was the earliest metric to show a supported breakpoint, after removal of only 0.46% of individuals but 30.1% of taxa. Taxa represented by few individuals thus had a disproportionate effect on estimated between-site differences when their occurrences were uneven. Mean Bray–Curtis dissimilarity and total Hellinger beta diversity responded later, as greater cumulative abundance was removed; *β*_sne_ had no supported breakpoint. This sequence indicates that changes in site identities were detected before strong shifts in abundance-weighted structure. Common and rare macroinvertebrates can differ in their contributions to beta diversity [39], while locally distinctive headwater assemblages contribute to regional variation [9]. Turnover is also a major component of beta-diversity change in other riverine assemblages [40,41].

Within a dendritic catchment, taxa with similar low regional abundance may occupy different parts of the stream network. Network position and connectivity shape the environmental and spatial context of these occurrences [10,11]. Taxa restricted to a few tributaries or transition zones can therefore have greater effects on site differentiation than their share of total individuals suggests. The response also depends on how taxa are removed: other removal processes can produce different turnover–nestedness patterns [42], and taxonomic resolution and dissimilarity measure influence the representation of rare records [4,43]. Because the data came from one dry-season survey, regional abundance and occupancy are best interpreted together with detection and seasonal phenology [44]. Reporting regional abundance, occupancy, and local abundance together makes these spatial patterns more informative for monitoring.

### 4.2. Reorganization of Environmental and Spatial Associations During Sequential Taxon Removal

The combined model consistently explained more variation than either the environmental or spatial model alone, indicating that both predictor sets captured structure in the observed community matrix. The environmental model generally explained more variation than the spatial model, while the positive pure spatial fraction indicated a spatial pattern not shared with the measured environmental gradients. The combined-model breakpoint at removal position 27 occurred when only 0.63% of individuals had been removed. Thus, records with low regional abundance contributed information relevant to the fitted environmental and spatial associations [13,45].

Elevation and stream width represented reach position and habitat scale; dissolved oxygen, redox potential, and electrical conductivity described complementary aspects of water chemistry and local conditions. Taxa concentrated along these gradients can strengthen the measured environmental association, whereas the spatial component can reflect network geometry, connectivity, and spatially structured conditions. Watershed and fluvial meta-ecosystem perspectives provide a framework for these linked local and network-scale patterns [46,47,48,49]. The changing variation fractions show that sequential removal altered how the observed assemblages corresponded to the fixed environmental and spatial predictors.

### 4.3. Nonlinear Response of Community Compositional Retention

Cumulative CSI declined nonlinearly, with a supported breakpoint at removal position 51. Early removals had small effects on site abundance, but their contributions accumulated. Later removals eliminated taxa with greater abundance from matrices that had already lost earlier taxa, producing larger departures from each site’s complete assemblage. The breakpoint therefore identifies a part of the fixed filtering sequence at which compositional similarity became more sensitive to further removal. Context-dependent biodiversity responses and variation across spatial scales have also been documented in other systems [16,17,18,50,51,52]; here, the relevant context is the abundance structure remaining at each removal step.

Variation in CSI among sites widened late in the sequence. A taxon with low regional abundance may still contribute a large share of individuals at a particular site, so its removal has a larger local effect there than at sites where it is absent. As removals accumulated, differences in richness, dominance, and co-occurrence produced an increasingly wide range of site-level CSI values. The regional mean described the overall trajectory, while the quantiles identified sites with greater compositional departure. The early turnover response and widening CSI range together show how uneven taxon distributions shape both between-site differentiation and each site’s similarity to its complete assemblage. Long-term stream studies likewise emphasize the importance of taxon identity and local context for community variation [53].

### 4.4. Interpretation of Taxon-Specific Effects

Isolated and sequential marginal CSI losses had nearly identical taxon rankings, consistent with the strong influence of abundance on both measures. Sequential marginal loss was usually greater because the same absolute removal represented a larger share of the remaining community later in the sequence. *Nemoura* sp. had the greatest isolated CSI loss, whereas *Cryptoperla* sp. had the greatest sequential marginal loss. These rankings show that a taxon’s measured effect depends on the abundance structure against which its removal is evaluated. The broader effect profiles also differed across composition, environmental and spatial associations, and CSI, reflecting differences in observed abundance and distribution among taxa.

Taxa with greater local abundance mainly affected within-site compositional similarity, while taxa with uneven spatial distributions had larger effects on turnover and fitted environmental or spatial associations. The three effect dimensions therefore conveyed complementary information about the observed matrix. Evidence from other ecosystems likewise shows that low-abundance taxa can differ in their functional contributions [54,55,56]. Retaining taxon identities and occurrence patterns alongside abundance improves the interpretation of filtering sensitivity. Chironomidae comprised 28.63% of individuals, primarily *Orthocladiinae* spp. (27.63%; 53 of 54 sites). Subfamily-level identification may conceal rare genera and shift abundance ranks or breakpoint positions under finer resolution. These taxon-specific results therefore apply to the observed mixed taxonomic resolution.

### 4.5. Implications for Monitoring and Analytical Filtering

Aquatic insect monitoring data should retain raw counts and report regional relative abundance, number of occupied sites, and local abundance together. Low-abundance records should not be removed with a single abundance threshold. For community comparisons or ecological assessment, the complete and filtered matrices should be analyzed in parallel and threshold sensitivity should be tested. Sampling gear, taxonomic resolution, and metric selection affect assessment results, and biodiversity monitoring and ecological-condition assessment require different data-processing schemes [8].

Sites and taxa with large compositional changes after filtering provide useful targets for follow-up sampling and taxonomic verification. This uses sensitivity analyses to guide data validation and monitoring design while leaving conservation decisions to assessments that include additional ecological evidence.

## 5. Conclusions

Low-abundance taxa are not negligible background information. Their uneven distributions contribute to estimated spatial turnover and produce effects on environmental and spatial associations and community compositional retention that change along the sequential-removal trajectory, while the main effect dimension differs among taxa. Monitoring and community analyses in mountain streams should retain original taxon records when possible; set filtering rules according to detection reliability, spatial distribution, taxonomic resolution, and study objectives; and test robustness through threshold-sensitivity analysis and comparison with the complete matrix. These inferences concern low-abundance taxon records at the observed mixed taxonomic resolution.

## Data Availability

Raw abundance, environmental, site-coordinate, and taxonomic data are provided in Supplementary File S1.xlsx. The derived statistical summaries for Figure 3, Figure 4, Figure 5 and Figure 6 are provided in S2.xlsx.

## Supplementary Materials

The following supporting information can be downloaded at https://www.mdpi.com/article/10.3390/insects17101007/s1. S1.xlsx (Table S1: Site coordinates, abundance, taxonomy, and environmental measurements in four worksheets); S2.xlsx (Table S2a–d: Concise statistical summaries for Figure 3, Figure 4, Figure 5 and Figure 6).
